# Effects of artificial cycles with and without gonadotropin-releasing hormone agonist pretreatment on frozen embryo transfer outcomes in patients with adenomyosis

**DOI:** 10.1038/s41598-021-98918-5

**Published:** 2021-09-29

**Authors:** Muzi Li, Lihong Xu, Heng Zhao, Yanbo Du, Lei Yan

**Affiliations:** 1 Center for Reproductive Medicine, Cheeloo College of Medicine, Shandong University, Jinan, People’s Republic of China; 2 Center for Reproductive Medicine, Shandong Provincial Hospital Affiliated to Shandong First Medical University, Jinan, 250021 Shandong People’s Republic of China; 3 National Research Centre for Assisted Reproductive Technology and Reproductive Genetics, Jinan, People’s Republic of China; 4 Key Laboratory of Reproductive Endocrinology at Shandong University, Ministry of Education, Jinan, People’s Republic of China

**Keywords:** Reproductive disorders, Infertility, Clinical trial design

## Abstract

Gonadotropin-releasing hormone agonist (GnRH-a) is generally added to the improve pregnancy outcomes of hormone replacement therapy cycles among patients with adenomyosis. We aimed to investigate whether adding GnRH-a can result in better pregnancy outcomes. This retrospective analysis included 341 patients with adenomyosis who underwent frozen embryo transfer (FET) after in vitro fertilization (IVF). The control group was treated only with hormone replacement therapy cycles to prepare the endometrium, and GnRH-a was added to the study group before hormone administration to adjust the menstruation cycle. Based on the similar baseline values and embryological data, there was no significant difference in the clinical pregnancy rates (40.63% vs. 42.54%, *P* = 0.72) and live birth rates (23.75% vs. 23.75%, *P* = 0.74) of the control and study groups. Other secondary outcomes, including the rates of clinical miscarriage, ectopic pregnancy, preterm birth and term birth, were not significantly different between the two groups. Compared with the hormone replacement therapy cycle alone, GnRH-a downregulation based on a hormone replacement therapy cycle may not increase the rate of clinical pregnancy or live birth of IVF-ET with FET among infertile patients with adenomyosis.

## Introduction

Adenomyosis is a condition in which endometrium-like epithelial and stromal tissues are present outside the endometrium and invade into the myometrium. Some studies have shown that patients with adenomyosis have lower implantation rates, clinical pregnancy rates and ongoing pregnancy rates but higher miscarriage rates than patients without adenomyosis^1,2^. At the same time, patients with adenomyosis benefit less from in vitro fertilization (IVF)/intracytoplasmic sperm injection (ICSI)^3,4^. Considering the adverse effects of adenomyosis, many protocols have been attempted to improve reproductive outcomes.

Gonadotropin-releasing hormone (GnRH) secreted by the hypothalamus can stimulate the pituitary to secrete FSH and LH, which in turn stimulates the ovaries to secrete sex hormones, forming the hypothalamic-pituitary-ovarian regulatory system. Because the affinity of gonadotropin-releasing hormone agonist (GnRH-a) with the receptor is much higher than that of GnRH, and it is not easy to be degraded, the complex formed is not easy to deconstruct, resulting in the reduction of receptor sites and receptor downregulation. GnRH-a reduces the sensitivity of the pituitary gland, reduces or inhibits the appearance of spontaneous LH peaks, and avoids spontaneous ovulation.

Whether GnRH-a downregulation can help patients with endometriosis have good reproductive outcomes is still disputed. A meta-analysis suggested that GnRH-a can effectively elevate the clinical pregnancy rates of patients with endometriosis^5^. In assisted reproductive technology (ART), GnRH-a has been widely used in patients with endometriosis for frozen or fresh embryo transfer to improve pregnancy outcomes^6–9^. Another study found that, regarding fresh and frozen embryo transfer (FET), GnRH-a was beneficial only for fertilization rates but not clinical pregnancy rates among women with endometriosis^10^. Additionally, two studies indicated that GnRH-a downregulation did not work well for patients with endometriosis undergoing IVF^11,12^.

In the context of adenomyosis, GnRH-a has been used for pituitary downregulation to improve reproductive conditions in ART. Several studies have shown the potential efficacy of adding GnRH-a to the treatment course in terms of pregnancy outcomes among women who undergo IVF/ICSI, and they support the use GnRH-a downregulation to improve the success rates of IVF/ICSI involving fresh embryo transfer or FET^13–15^.

However, considering the controversial role of GnRH-a in patients with endometriosis, we analyzed pregnancy outcomes to determine whether GnRH-a downregulation would be beneficial in endometrial preparation protocols for adenomyosis with FET based on hormone replacement therapy cycles.

## Materials and methods

We analyzed the data from the hospital electronic database for patients undergoing FET between 2013 and 2018. This retrospective cohort study was conducted at the Reproductive Hospital Affiliated with Shandong University. The inclusion criteria were patients who were no more than 45 years old and were diagnosed with adenomyosis mainly by two-dimensional ultrasound, which revealed (1) subjective enlargement of the uterine corpus, (2) an asymmetrically thickened myometrium between the anterior and posterior walls, (3) heterogeneity of the myometrium/hypoechoic striations, and (4) poor definition of the endometrial-myometrial junction^16^. In this study, only the first FET cycle was analyzed. The stage of the embryo transferred was day 3, day 5 or day 6, and the number of transferred embryos was no more than two. Patients with donor oocytes were excluded. Other exclusion criteria were malformations of the reproductive system without therapy, hydrosalpinx, polycystic ovary syndrome (PCOS), endometriosis, malignant diseases of the reproductive system and a chromosomal abnormality or disease-associated gene in one member of the couple. The patients in group A were received dual treatment with the hormone replacement cycle and GnRH-a, and group B received treatment with the hormone replacement cycle.

Vitrification freezing and thawing operation steps: at room temperature, the early embryos were placed in ES (Equilibration Solution) medium for 5–10 min. After a waiting period to allow the embryos to shrink and re-expand, the embryos were moved into the VS medium for 30–40 s, and the carrier was quickly loaded. The excess liquid (with only 1–2 µl liquid) was sucked off directly into liquid nitrogen. After the tube was installed, it was placed in a liquid nitrogen tank for storage. During thawing, the carrier containing the embryo was removed from the liquid nitrogen, immediately placed it into a 1.0 M sucrose solution at 37 °C and incubated for 1.5–2 min until a clear outline of the embryo's blastomere could be observed. Then, the mixture was moved into 0.5 M, 0.25 M, and 0 M sucrose solutions at room temperature to be equilibrated for 3–5 min. In the final 0 M sucrose solution, the mixture was warmed to 37 °C and allowed to equilibrate for 5 min. Finally, the embryo culture medium was transferred to an incubator for culture. The recovery rate of 8-cell embryos obtained by this method was over 95%.

### Procedures

For the hormone replacement therapy cycle regimen, the endometrium prepared with oral estradiol valerate at a dose of 4 mg daily was started on days 2–4 of the menstrual cycle for 5–6 days and then 6 mg for the following 5–6 days. Endometrial thickness was monitored by transvaginal ultrasound after 10–12 days of medication, along with the serum levels of FSH, LH, estradiol (E2) and progesterone (P). Thereafter, the dose of estradiol valerate, which was 8 mg/d maximally, was modulated according to the endometrial thickness and E2 levels of the patient. P capsule 200 mg/day and oral dydrogesterone 40 mg/day as luteal phase support were added when the endometrial thickness reached 7 mm or more and FET was carried out 6 days later. Estradiol valerate at the dose for endometrial preparation was continued until the day of the serum hCG test, which was 2 weeks after the embryo transfer. If pregnancy was achieved, estradiol valerate was stopped gradually at 7–8 weeks of gestation, and P capsule and oral dydrogesterone were continued until 12 weeks of gestation.

In the study group, GnRH-a long-acting injection including triptorelin and leuprorelin was used at a dose of 3.75 mg during menstruation. After a follow-visit 30 days later, patients started their hormone replacement cycles. Some patients had a larger uterus due to adenomyosis and received multiple GnRH-a injections at the 3.75-mg dose once per month.

In addition, some patients carried out a fresh emryo transfer before FET. After routine ovulation stimulation programs including long GnRH-a protocols, short GnRH-a protocols, ultra-long GnRH-a protocols and GnRH-a antagonist protocols, patients will conduct a fresh embryo transfer in 3 days.

### Outcome measures

One primary outcome of this study is clinical pregnancy rates and live birth rates. Secondary outcomes include clinical miscarriage rates, ectopic pregnancy rates, preterm pregnancy rates and term pregnancy rates. Clinical pregnancy is defined as a pregnancy diagnosed by ultrasonographic visualization of one or more gestational sacs or definitive clinical signs of pregnancy. Apart from intrauterine pregnancy, it includes a clinically documented ectopic pregnancy. Live birth is defined as the complete expulsion or extraction from a woman of a product of fertilization after 22 completed weeks of gestational age, which, after such separation, breathes or shows any other evidence of life, such as heartbeat, umbilical cord pulsation or definite movement of voluntary muscles, irrespective of whether the umbilical cord has been cut or the placenta is attached. A birth weight of 500 g or more can be used if gestational age is unknown. Live births refer to the individual newborn; for example, a twin delivery represents two live births. Secondary outcomes were defined according to our previously published paper^17^.

### Statistical analysis

Relevant data were analyzed by SPSS 22.0. *P* < 0.05 was considered to indicate statistical significance. Continuous variables with normal distributions are presented as the mean ± standard deviation and were analyzed by means of the two-sample t-test. All variables were tested for normality. Categorical variables are presented as percentages, and inter-group comparisons were carried out by means of the chi-square test or Fisher’s exact test. Binary logistic regression was used to analyze the relationship between the dichotomous dependent variable and independent variable.

### Ethics approval

The study protocol was approved by the Ethics Committee of the Reproductive Hospital Affiliated with Shandong University and adhered to relevant ethical guidelines. The studies involving human participants were reviewed and approved by the research ethics committee of Reproductive Hospital affiliate d to Shandong University. The patients/participants provided their written informed consent to participate in this study.

## Results

In total, 341 women were analyzed in this study. One hundred and sixty females in Group A underwent GnRH-a downregulation treatment based on the hormone replacement therapy cycle, and the other one hundred eighty-one patients in Group B were treated only with hormone replacement therapy. In Group A, 73.75% and 15.625% of patients respectively underwent once and twice GnRH-a down-regulation and only 10.625% of patients underwent no less than three times GnRH-a down-regulation.

The baseline values of the patients are presented in Table 1. There was no significant difference in age, height, weight or body mass index (BMI) (34.56 ± 4.49 vs. 35.25 ± 4.95, *P* = 0.18; 162.63 ± 5.57 cm vs. 161.56 ± 5.29 cm, *P* = 0.08; 64.99 ± 11.04 kg vs. 63.76 ± 9.42 kg, *P* = 0.27; 24.56 ± 3.87 vs. 24.43 ± 3.42, *P* = 0.73) between the two groups. Types of infertility and baseline hormone values, including FSH, LH, E2 and PRL, were comparable between the two groups (6.54 ± 1.89 IU/L vs. 6.32 ± 2.04 IU/L, *P* = 0.31; 4.39 ± 2.05 IU/L vs. 4.85 ± 2.57 IU/L, *P* = 0.07; 42.38 ± 33.86 pg/ml vs. 48.08 ± 76.54 pg/ml, *P* = 0.39; 19.87 ± 35.15 ng/ml vs. 16.76 ± 19.48 pg/ml, *P* = 0.31). The percentages of primary and secondary infertility, duration of infertility, and causes of infertility mainly including female factors, male and female factors and unknown factors were also comparable between the two groups (40.62% vs. 33.70%, 59.38% vs. 66.30%, *P* = 0.19; 4.07 ± 3.27 vs. 4.29 ± 3.45, *P* = 0.55; 91.25% vs. 93.89%, 6.88% vs. 3.87%, 1.87% vs. 2.74%, *P* = 0.41). The endometrial thickness of patients in group B was lower than that in Group A (0.96 ± 0.20 cm vs. 0.91 ± 0.18 cm, *P* = 0.02), but the mean values were both above 7 mm and the difference did not have clinical significance^18^.

**Table 1:** Comparison of baseline data of the two groups.

	GROUP A (n = 160)^a^	GROUP B (n = 181)^b^	Two-tailed *P* value
	Mean (SD)	Mean (SD)	
Age (SD) (year)	34.56 (4.49)	35.25 (4.95)	0.18
Height (SD) (cm)	162.63 (5.57)	161.56 (5.29)	0.08
Weight (SD) (kg)	64.99 (11.04)	63.76 (9.42)	0.27
BMI (SD)	24.56 (3.87)	24.43 (3.42)	0.73
**Laboratory tests**			
FSH (SD) (IU/L)	6.54 (1.89)	6.32 (2.04)	0.31
LH (SD) (IU/L)	4.39 (2.05)	4.85 (2.57)	0.07
E2 (SD) (Pg/ml)	42.38 (33.86)	48.08 (76.54)	0.39
PRL (SD) (ng/ml)	19.87 (35.15)	16.76 (19.48)^c^	0.31
**Infertility**			0.19
Primary infertility—no./total no. (%)	65/160 (40.62)	61/181 (33.70)	
Secondary infertility—no./total no. (%)	95/160 (59.38)	120/181 (66.30)	
Endometrial thickness on hCG trigger day (cm)	0.96 (0.20)^d^	0.91 (0.18)	0.02
Duration of infertility (year)	4.07 (3.27)	4.29 (3.45)	0.55
Causes of infertility-no./total no. (%)			0.41
Female factors	146/160 (91.25)	169/181 (93.89)	
Male and female factors	11/160 (6.88)	7/181 (3.87)	
Unknown factors	3/160 (1.87)	5/181 (2.74)	

^a^In the study group (n = 160), GnRH-a was added based on GnRH-a before hormone administration to adjust the menstruation cycle.

^b^The control group (n = 181) was treated only with an artificial hormone cycle to prepare the endometrium.

^c^One value was missing.

^d^Five values were missing.

The embryological data are shown in the Table 2. The timing of embryo transfer was divided into two categories, namely, cleavage-stage embryo transfer and blastocyst-stage embryo transfer. Cleavage-stage embryos were defined as 2-cell-stage embryos up to, but not including, the morula stage. Blastocyst-stage embryos were defined as those whose preimplantation development that occurred around days 5–6 after insemination or ICSI. The blastocyst contained a fluid-filled central cavity (blastocoele), an outer layer of cells (trophectoderm) and an inner group of cells (inner cell mass). The number of transferred embryos was generally no more than two. The time of embryo transfer and the number of transferred embryos did not differ obviously between the two groups. The percentages of day-3, day-5 and day-6 embryo transfers and the percentages of one- and two-embryo in the two groups were not significantly different (1.25% vs. 2.76%, 72.5% vs. 75.14%, 26.25% vs. 22.10%, *P* = 0.44; 89.83% vs. 88.40%, 10.62% vs. 11.60%, *P* = 0.78). High-quality embryos of D3 were generally defined as embryos derived from normal fertilized eggs, and the number of embryonic cells on the third day after fertilization was 7–9, the cell size was in line with the developmental stage, the degree of fragmentation was less than 10%, and the embryo was not multinucleated. High-quality blastocysts were generally defined as blastocysts of stage 3 and above in the Gardner scoring standard, and the inner cell mass and trophoblast scores did not contain C. There was no significant difference in high-quality embryo rate of D3 and high-quality blastocyst ratio between the two groups (25.00% vs. 77.78%, *P* = 0.22; 70.52% vs. 74.61%, *P* = 0.38).

**Table 2:** Comparison of embryological characteristics of the two groups.

	GROUP A (n = 160)^a^	GROUP B (n = 181)^b^	Two-tailed *P* value
**Stage of embryo transferred—no./total no. (%)**			
D3	2/160 (1.25)	5/181 (2.76)	0.44
D5	116/160 (72.5)	136/181 (75.14)	
D6	42/160 (26.25)	40/181 (22.10)	
**No. of embryos transferred**			0.78
One embryo—no./total no. (%)	143/160 (89.38)	160/181 (88.40)	
Two embryos—no./total no. (%)	17/160 (10.62)	21/181 (11.60)	
**High-quality embryo rate**			
High-quality embryo rate of D3—no./no. of normally fertilized eggs. (%)	1/4 (25.0)	7/9 (77.78)	0.22
High-quality blastocyst ratio—no./total no. (%)	122/173 (70.52)	144/193 (74.61)	0.38

^a^In the study group (n = 160), GnRH-a was added based on GnRH-a before hormone administration to adjust the menstruation cycle.

^b^The control group (n = 181) was treated only with an artificial hormone cycle to prepare the endometrium.

Considering some participants may undergo a fresh embryo transfer before FET, the number of these patients were compared and the difference was not statistically significant, (53.13% vs. 52.49%, *P* = 0.91). At the same time, we analysised the reproductive outcomes of the fresh embryo transfer between the two groups. The GnRH-a downregulation group in clinical pregnancy rates, biochemical pregnancy rates, ectopic pregnancy rates, clinical miscarriage rates and live birth rates did not show any significant difference compared to the hormone replacement cycle group (30.59% vs. 24.21%, *P* = 0.34; 8.24% vs. 7.37%, *P* = 0.83; 73.08% vs. 56.52%, *P* = 0.23; 3.85% vs. 8.70%, *P* = 0.91; 8.24% vs. 10.53%, *P* = 0.60).

As shown in Table 3, we compared the pregnancy outcomes of the infertility patients with adenomyosis in the two groups. Among both groups, the clinical pregnancy rates (40.63% vs. 42.54%, *P* = 0.72) and the live birth rates (23.75% vs. 23.75%, *P* = 0.74) were not different, and the clinical miscarriage rates (41.5% vs. 44.2%, *P* = 0.754), biochemical pregnancy rates (13.75% vs. 11.05%, *P* = 0.45) and ectopic pregnancy rates (3.08% vs. 3.90%, *P* = 1) were similar.

**Table 3:** Comparison of clinical pregnancy outcomes between the two groups.

	GROUP A (n = 160)^a^	GROUP B (n = 181)^b^	Two-tailed *P* value
**Clinical outcome (%)**			
Clinical pregnancy	65/160 (40.63)	77/181 (42.54)	0.72
Biochemical pregnancy rate	22/160 (13.75)	20/181 (11.05)	0.45
Clinical miscarriage rate	27/65 (41.54)	34/77 (44.16)	0.75
Ectopic pregnancy	2/65 (3.08)	3/77 (3.90)	1^c^
Live birth rate	38/160 (23.75)	43/181 (23.75)	0.74
Preterm birth	5/160 (3.12)	5/181 (2.76)	0.84
Full-term birth	33/160 (20.63)	38/181 (20.99)	0.95

^a^In the study group (n = 160), GnRH-a was added based on GnRH-a before hormone administration to adjust the menstruation cycle.

^b^The control group (n = 181) was treated only with an artificial hormone cycle to prepare the endometrium.

^c^This value was calculated by means of Fisher’s exact test.

Binary logistic regression, shown in Table 4, revealed that BMI, endometrial thickness on the HCG trigger day or No. of high-quality embryos were not related to clinical pregnancy rates. However, it was the age and the embryo stage at transfer (OR 0.42, 95% CI 0.19 to 0.93 for patients patients from 31 to 35 years old compared with patients no more than 30 years old; OR 0.61, 95% CI 0.30 to 1.26 for patients from 36 to 40 years old compared with patients no more than 30 years old; OR 0.70, 95% CI 0.33 to 1.48 for patients from 41 to 45 years old compared with patients no more than 30 years old; OR 0.38, 95% CI 0.76 to 1.96 for women having a day-5 embryo transfer compared with women having a day-3 embryo transfer; OR 0.37, 95% CI 0.20 to 0.68 for women having a day-6 embryo transfer compared with women having a day-3 embryo transfer) that were the independent variables associated with clinical pregnancy rates. Redarding the live birth rates, binary logistic regression analysis, shown in Table 5, revealed that there was no association with BMI, endometrial thickness on the HCG trigger day, and No. of high-quality embryos. Age and the embryo stage at transfer were independent variables related to the live birth rates for patients with adenomyosis (OR 0.16, 95% CI 0.06 to 0.48 for patients patients from 31 to 35 years old compared with patients no more than 30 years old; OR 0.42, 95% CI 0.15 to 1.20 for patients from 36 to 40 years old compared with patients no more than 30 years old; OR 0.38, 95% CI 0.13 to 1.11 for patients from 41 to 45 years old compared with patients no more than 30 years old; OR 0.20 95% CI 0.03 to 1.32 for women having a day 5-embryo transfer compared with women having a day-3 embryo transfer; OR 0.26, 95% CI 0.11 to 0.59 for women having a day-6 embryo transfer compared with women having a day-3 embryo transfer).

**Table 4:** Binary logistic regression of influencing factors on pregnancy rates in the two groups.

	OR (95%CI)	Two-tailed *P* value
**Age (year)**		
≤ 30		0.17
31–35	0.42 (0.19–0.93)	0.03
36–40	0.61 (0.30–1.26)	0.18
41–45	0.70 (0.33–1.48)	0.35
**Stage of embryo transferred**		
DAY3		0.01
DAY5	0.38 (0.76–1.96)	0.25
DAY6	0.37 (0.20–0.68)	< 0.01

**Table 5:** Binary logistic regression of influencing factors on live birth rates in the two groups.

	OR (95%CI)	Two-tailed *P* value
**Age (year)**		
≤ 30		< 0.01
31–35	0.16 (0.06–0.48)	< 0.01
36–40	0.42 (0.15–1.20)	0.11
41–45	0.38 (0.13–1.11)	0.08
**Stage of embryo transferred**		
DAY3		0.01
DAY5	0.20 (0.03–1.32)	0.10
DAY6	0.26 (0.11–0.59)	< 0.01

The time of GnRH-a downregulation in the study group was counted. A total of 73.75% and 15.625% of patients underwent one and two GnRH-a downregulation treatments, respectively, and only 10.625% of patients underwent no less than three GnRH-a downregulation events.

## Discussion

In this retrospective study, there were no obvious effects of GnRH-a on the pregnancy outcomes of patients with adenomyosis. The comparable clinical pregnancy rates of the two groups seemed inconsistent with GnRH-a-mediated increase in the likelihood of becoming pregnancy among females with adenomyosis^13,14^. The preterm and full-term birth rates of the two groups were similar, which was in contrast to the findings of previous epidemiological studies^19^.The difference may be attributed to the difference in participants’ age, duration of infertility, type of down-regulation protocol used, number and quality of the transferred embryos, because these studied included in meta-analysis were heterogenous.

One meta-analysis explored the association between endometrial thickness and pregnancy outcomes and showed that a thinner endometrium (< 7 mm) resulted in low ongoing pregnancy rates and live birth rates^20,21^. In this study, the difference in endometrial thickness between the two groups was statistically significant; however, the mean endometrial thickness values of both groups were above 9 mm. Therefore, it is debatable whether the different endometrial thicknesses impaired pregnancy outcomes in this study.

One randomized controlled trial indicated that embryo transfer conducted with a day-5 embryo was more likely to result in a higher ongoing or cumulative pregnancy rate than transfer with a day-3 embryo^22^. Another meta-analysis demonstrated that clinical pregnancy rates and live birth rates were significantly higher following day-5 blastocyst transfers than day-6 blastocyst transfers^23^. Both Group A and Group B had patients with day-3, day-5 and day-6 of embryo transfers, and the variation between the two groups was not statistically significant even if the proportion of day-3 embryos was relatively low. The vast majority of embryo transfers in both groups involved day-5 embryos, which can improve the pregnancy rate.

The proportion of patients with primary infertility in Group A was higher than that in Group B, although the difference in the ratio of primary to secondary infertility between the two groups was not statistically significant. Studies have demonstrated that the pregnancy rate of patients with secondary infertility is significantly higher than that of patients with primary infertility^24,25^. The higher percentage of primary infertility patient in Group A may be a key factor in decreasing rates.

Regarding the number of transferred embryos, one study indicated that two elective single-embryo transfers can achieve better reproductive outcomes than one double-embryo transfer when blastocysts are used^26^. In our study, the single-embryo transfer protocol was also regarded as the best protocol to ensure good pregnancy outcomes. In addition, it was noticeable that the total clinical pregnancy and live birth rates of patients remained low regardless of the measures taken Because adenomyosis itself will cause serious adverse effects on pregnancy outcomes as previous study suggested, including impaired endometrium-myometrium interface, altered uterine peristaltic activity and so on^1,19^.

In this study, GnRH-a did not have an obvious effect in terms of improving pregnancy outcomes. We came up with several reasons to explain this phenomenon. First, the current cycles of GnRH-a were not sufficient to produce the effects of the drug needed to be effective in patients with adenomyosis. Second, we did not obtain information on the degree of adenomyosis among patients in the two groups. It is possible that many of the patients did not suffer from severe adenomyosis, which may be a contributor to the negative results in terms of GnRH-a-mediated improvements in pregnancy outcomes. Finally, is it possible that patients were not sensitive to GnRH-a, which would result in the uterus not changing much, or that GnRH-a treatment did not work at all.

One advantage of our study was that the number of women with adenomyosis was relatively high, as the study included patients from a time span of 6 years. Additionally, according to the 2018 specialist consensus published in the Journal of Reproductive Medicine, the number of embryos transferred did not exceed two over a 40-year period, and single-embryo transfer was recommended. In our study, single-embryo transfer constituted the vast majority of embryo transfers, and the others were double-embryo transfers, which was consistent with the consensus. These two advantages made this study applicable to a larger population with adenomyosis. Furthermore, we found that good reproductive outcomes remained at a low level, despite different measures being taken, which means that many future studies are needed to improve the reproductive outcomes of women with adenomyosis.

There were two obvious limitations in this study. Firstly, if patients could get enough duration of GnRH-a downregulation, the attachment of high quality embryos may be affected as most of our patients only underwent once GnRH-a downregulation. The severity of adenomyosis, which may influence the therapeutic effect, was not taken into account. These questions both need to be studied in the future.

Although many studies have suggested that administrating GnRH agonists is beneficial for patients with adenomyosis in terms of improving reproductive outcomes of IVF-ET, our study did not demonstrate any advantages for the addition of GnRH agonists based on the hormone replacement therapy cycle. Similar negative findings were reported in endometriosis patients^10–12^. In addition, Movahedi et al. performed a study in which endometrial preparation for FET using GnRH agonists appeared to be as effective as FET without these agonists^27^. The other two articles also did not suggest that GnRH-a downregulation had any significant advantages for IVF-ET^28,29^. These results provide some supportive evidence for our study results.

In conclusion, adenomyosis patients with or without GnRH-a downregulation based on the hormone replacement therapy cycle had similar reproductive outcomes. Adding GnRH-a based on the hormone replacement therapy cycle may not increase the rate of clinical pregnancy or live birth.

## Data Availability

Data and material are available. The datasets generated and/or analyzed during the current study are available by request.

## Code availability

Software application was available.
